# Supplementary material for: TR-107, a novel mitochondrial ClpP agonist, induces robust antitumor activity against preclinical models of adrenocortical carcinoma
Source: bioRxiv. 2026 Aug 11:2026.08.10.743339. Preprint. [Version 1] doi: 10.64898/2026.08.10.743339 (PMC13640636; doi:10.64898/2026.08.10.743339)
Supplement: Supplementary file 1 [file media-1.pdf]

Supplementary Figure S1

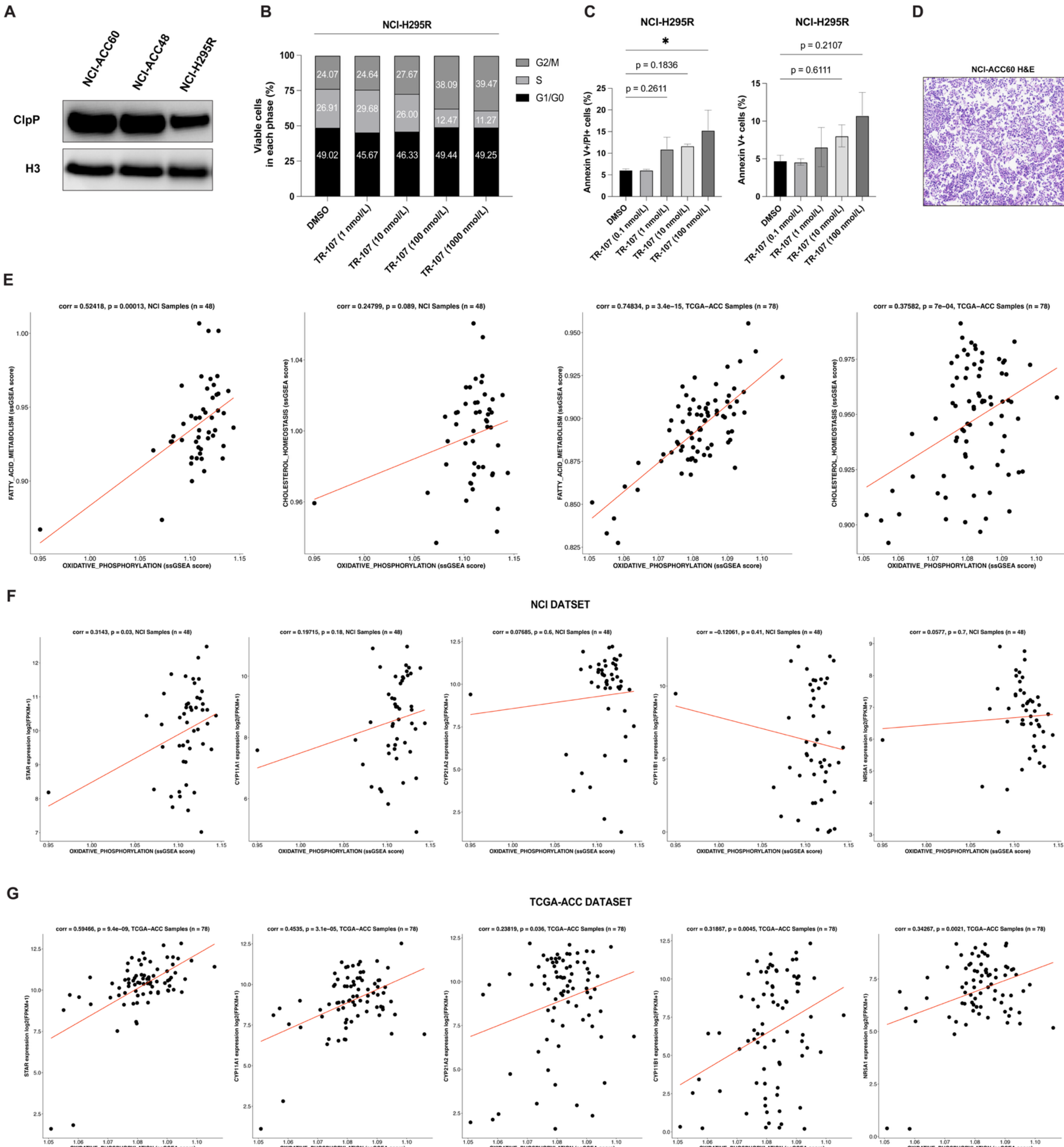

A, Immunoblot analysis of ClpP expression in representative ACC models NCI-H295R, NCI-ACC48 PDOs, and NCI-ACC60 PDOs. **B,** Fractions of viable NCI-H295R cells in each cell cycle phase following 48-hour TR-107 treatment. **C,** Quantification of Annexin V-positive/propidium iodide-positive and Annexin V-positive NCI-H295R cells following 48-hour TR-107 treatment. **D,** Hematoxylin & eosin (H&E) staining of NCI-ACC60 PDOs. **E,** Pearson correlation analyses between fatty acid metabolism, cholesterol homeostasis ssGSEA enrichment scores. **F** and **G,** the mRNA expression of key steroidogenic proteins relative to oxidative phosphorylation ssGSEA enrichment scores in NCI-ACC (**F**) and TCGA-ACC (**G**) tumor specimens. n.s., not significant; *, $P < 0.05$.

Supplementary Figure S2.

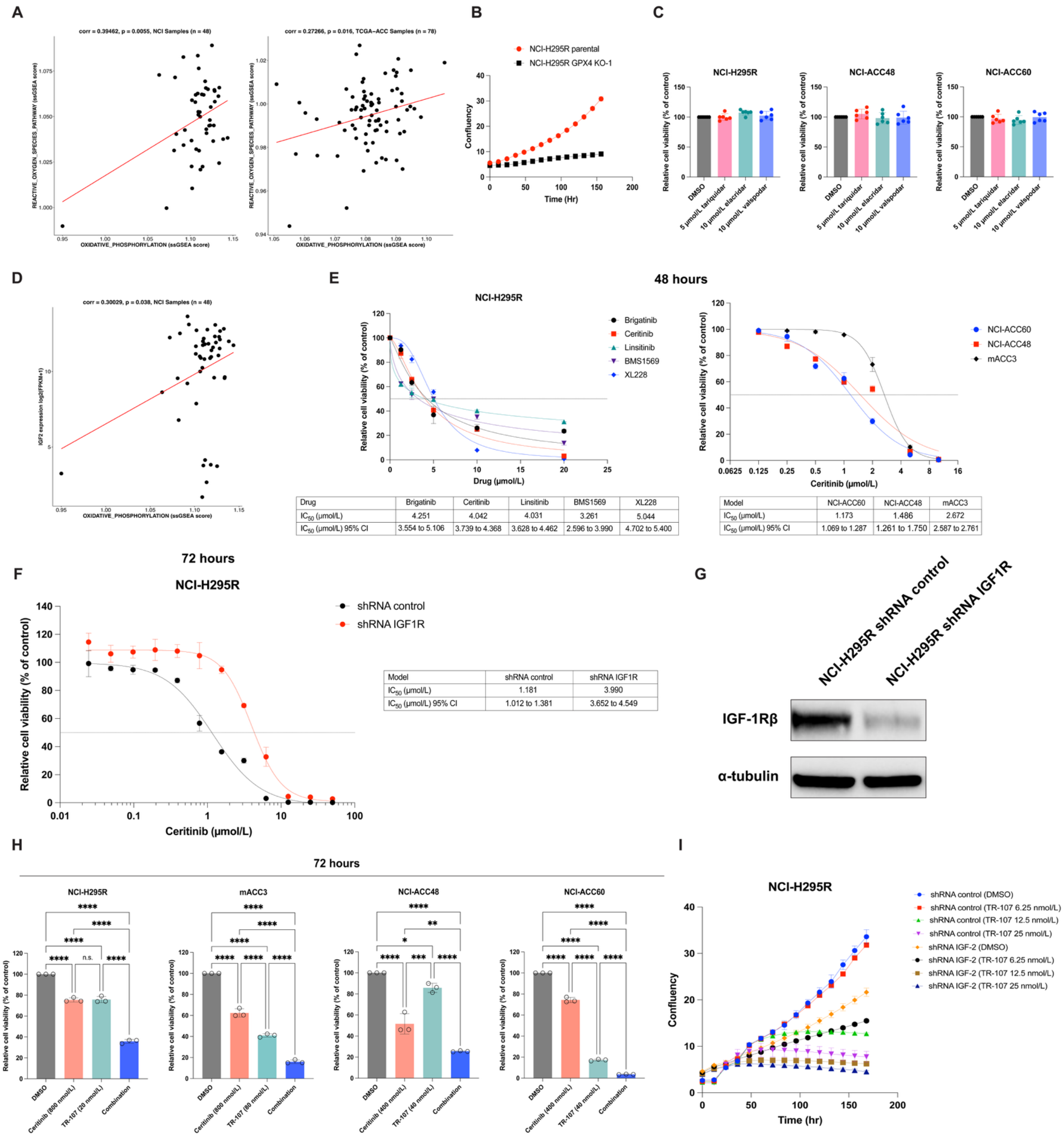

Drug	Brigatinib	Ceritinib	Linsitinib	BMS1569	XL228
IC_{50} (μmol/L)	4.251	4.042	4.031	3.261	5.044
IC_{50} (μmol/L) 95% CI	3.554 to 5.106	3.739 to 4.368	3.628 to 4.462	2.596 to 3.990	4.702 to 5.400

Model	NCI-ACC60	NCI-ACC48	mACC3
IC_{50} (μmol/L)	1.173	1.486	2.672
IC_{50} (μmol/L) 95% CI	1.069 to 1.287	1.261 to 1.750	2.587 to 2.761

Model	shRNA control	shRNA IGF1R
IC_{50} (μmol/L)	1.181	3.990
IC_{50} (μmol/L) 95% CI	1.012 to 1.381	3.652 to 4.549

A, Pearson correlation analysis between reactive oxygen species (ROS) ssGSEA enrichment scores and oxidative phopshorylation ssGSEA enrichment score in NCI-ACC and TCGA-ACC tumor specimens. **B,** Longitudinal basal cell proliferation of NCI-H295R GPX4 KO cells compared to NCI-H295R parental cells. Data reported as mean ± SEM, *n* = 3 technical replicates. **C,** Relative cell viability of NCI-H295R cells cultured with ABC transporter inhibitors tariquidar (5 µmol/L), elacridar (10 µmol/L), and valspodar (10 µmol/L) alone. Data reported as mean ± SD, *n* = 6 technical replicates. **D,** Pearson correlation analysis between *IGF2* expression and oxidative phopshorylation ssGSEA enrichment score in NCI-ACC and TCGA-ACC tumor specimens. **E,** Relative cytotoxic effects of variable IGF-1R inhibitors following 48-hour treatment in NCI-H295R, mACC3, NCI-ACC48, and NCI-ACC60 models. Data reported as mean ± SD, *n* = 3 technical replicates. **F,** Relative cytotoxic effects of 72-hour ceritinib treatment on NCI-H295R *IGF1R* shRNA and control shRNA cells. **G,** Immunoblot validation of NCI-H295R *IGF1R* shRNA and control shRNA cells. **H,** Relative cytotoxic effects of TR-107 plus ceritinib combinatory treatment in NCI-H295R, mACC3, NCI-ACC48, and NCI-ACC60 models following 72-hour treatment. Data reported as mean ± SD, *n* = 3 technical replicates. **I,** Longitudinal cell proliferation of NCI-H295R *IGF2* shRNA and NCI-H295R control shRNA cells following treatment with variable concentrations. Data reported as mean ± SEM, *n* = 3 technical replicates. n.s., not significant; *, $P < 0.05$; **, $P < 0.01$; ***, $P < 0.001$; ****, $P < 0.0001$.
